# Differential impact of ageing on cellular and humoral immunity to a persistent murine γ-herpesvirus

**DOI:** 10.1186/1742-4933-7-3

**Published:** 2010-02-02

**Authors:** Eric J Yager, In-Jeong Kim, Michael L Freeman, Kathleen G Lanzer, Claire E Burkum, Tres Cookenham, David L Woodland, Marcia A Blackman

**Affiliations:** 1 Center for Immunology & Microbial Disease, Albany Medical College, 47 New Scotland Avenue, Albany, NY, 12208, USA; 2 Pfizer Canada, Inc, Vaccine Research, 340 Terry Fox Drive, Suite 200, Kanata, ON K2K 3A2, Canada; 3 Trudeau Institute, 154 Algonquin Ave, Saranac Lake, NY 12983, USA

## Abstract

**Background:**

Oncogenic γ-herpesviruses establish life-long infections in their hosts and control of these latent infections is dependent on continual immune surveillance. Immune function declines with age, raising the possibility that immune control of γ-herpesvirus infection becomes compromised with increasing age, allowing viral reactivation and/or increased latent load, both of which are associated with the development of malignancies.

**Results:**

In this study, we use the experimental mouse γ-herpesvirus model, γHV68, to investigate viral immunity in aged mice. We found no evidence of viral recrudescence or increased latent load in aged latently-infected mice, suggesting that effective immune control of γ-herpesvirus infection remains intact with ageing. As both cellular and humoral immunity have been implicated in host control of γHV68 latency, we independently examined the impact of ageing on γHV68-specific CD8 T cell function and antibody responses. Virus-specific CD8 T cell numbers and cytolytic function were not profoundly diminished with age. In contrast, whereas ELISA titers of virus-specific IgG were maintained over time, there was a progressive decline in neutralizing activity. In addition, although aged mice were able to control de novo acute infection with only slightly delayed viral clearance, serum titers of neutralizing antibody were reduced in aged mice as compared to young mice.

**Conclusion:**

Although there is no obvious loss of immune control of latent virus, these data indicate that ageing has differential impacts on anti-viral cellular and humoral immune protection during persistent γHV68 infection. This observation has potential relevance for understanding γ-herpesvirus immune control during disease-associated or therapeutic immunosuppression.

## Background

Ageing impacts many aspects of mammalian biology, including immune function [1]. Immunological ageing is associated with a decline in the production of naïve T and B cells, defects in the production of high-affinity antibodies, and impaired CD4 T cell function [2-5]. As a consequence, the elderly exhibit a reduced responsiveness to vaccination and an increased susceptibility to newly encountered pathogens. Although not thoroughly studied, there are also data to suggest that ageing may dampen immune control over chronic viral infections. For example, the increased incidence of herpes zoster disease in the elderly is believed to be due in part to the waning of cell-mediated immune control over dormant varicella (chicken pox) virus reactivation [6].

The human γ-herpesviruses, Epstein-Barr virus (EBV) and Kaposi's sarcoma-associated herpesvirus (KSHV), are important pathogens that establish life-long latency in infected individuals and are associated with a wide variety of malignancies, including Burkitt's lymphoma, Hodgkin's disease, nasopharyngeal carcinoma, Kaposi's sarcoma, and B cell lymphoproliferative syndromes [7]. Most of the malignancies develop after years of viral dormancy, and are accompanied or triggered by viral reactivation [8]. An important role for immune control in preventing the development of malignancies is illustrated by the fact that immunosuppression, as a consequence of disease or suppressive immunotherapy, leads to the development of EBV-associated lymphoproliferative syndromes and lymphomas, and KSHV-associated Kaposi's sarcoma [8,9]. It is difficult to directly assess the age-associated oncogenic consequences of diminished immune control of the γ-herpesviruses, as the development of malignancies associated with γ-herpesvirus infection is a multistep process.

In order to directly assess the impact of ageing on the ability to maintain control of the γ-herpesviruses, we have employed the well-characterized, experimental murine γ-herpesvirus infection model, in which we can kinetically monitor several aspects of immune function. Murine γ-herpesvirus γHV68 (MHV-68 or murid herpesvirus-4) has significant structural and biological similarities to the two human herpesviruses, EBV and KSHV, and is emerging as an important experimental model for studying basic mechanisms of immune control of γ-herpesviruses in an easily manipulated animal system [10-14]. Intranasal administration of γHV68 to mice establishes an acute lytic infection in lung epithelial cells, which is normally controlled by day 13 postinfection via the anti-viral activities of CD4 and CD8 T cells [11,12]. Latency is established in the lung, concurrent with the lytic infection [15], and is subsequently established in splenic B cells, macrophages and dendritic cells [16-18]. Latent virus persists for the lifetime of the host, and is kept from reactivating to produce detectable levels of lytic virus by both cellular and humoral mechanisms of immune control [11,12,19]. Constant immunosurveillance is critical, as immunosupression leads to recrudescence of lytic virus in γHV68-infected mice.

In the current study we experimentally infected C57BL/6 mice intranasally with low doses of γHV68 and monitored immune control of the virus over time. Specifically, we assessed latent load, protection against re-infection, and virus-specific humoral and cellular immunity with increasing time after infection to determine the impact of ageing on immune control of a latent infection established during youth. We also examined the ability of aged mice to control a de novo γ-herpesvirus infection. The data reveal no evidence of viral recrudescence, or increase in latent viral load, with ageing. In addition, aged mice were capable of clearing lytic virus following de novo γHV68 infection with only slightly delayed kinetics. However, ageing had a differential impact on the cellular and humoral components of immune control. Whereas there was no overall reduction in virus-specific T cell numbers or function with age, and virus-specific antibody titers were found to remain stable, we observed a gradual decline in the neutralizing activity of serum taken from aged latently-infected mice. In addition, antibodies generated in aged mice following a de novo infection had impaired neutralizing activity as compared to antibodies generated in younger mice. Thus, although cellular immunity appears to be sufficient for the control of latent γHV68 infection throughout the life of the host, impaired humoral immunity, as observed with ageing, may have significant implications for maintaining immune control over γ-herpesvirus infection following therapeutic or infection-mediated immunosuppression.

## Results

### Viral latency is controlled in aged mice

**Figure 1:**
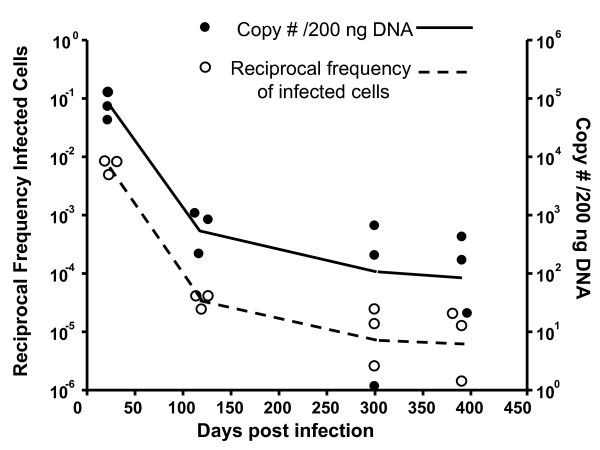
**γHV68 latency in the spleen remains stable with age**. The reciprocal frequency of latently-infected cells as determined by LDA/PCR is plotted on the left axis (open circles) and the genome copy number per 200 ng DNA is plotted on the right axis (closed circles) over time after intranasal infection with 400 PFU γHV68. The lines join the means of three individual mice at each timepoint.

As a first step in determining whether there is an age-associated decline in immune control of the persistent γ-herpesviruses, we monitored latent viral load in γHV68-infected mice over time following infection. We used two independent assays- the limiting dilution PCR assay (LDA/PCR) which allows determination of the frequency of latently-infected cells [19,20] and the quantitative PCR assay which allows determination of genome copy number within a standard amount of genomic DNA [21]. Latency assessed by either method was shown to decline after the early peak of latency in the spleen and then stabilize for more than a year after infection (Figure 1). Consistent with the stable latent load in aged mice, we failed to detect recrudescent lytic virus in the lung, spleen or a variety of other anatomical sites (data not shown). These data suggest that immune control over γHV68 latency is maintained with age.

### Protective immunity is maintained in aged mice

**Figure 2:**
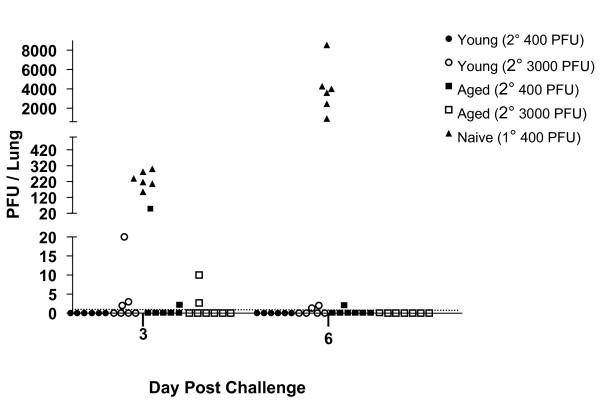
**Latently-infected aged mice are protected against re-infection by γHV68**. Young mice (3 months pi; circles) and aged mice (19 months pi; squares) were intranasally challenged with γHV68 (400 PFU, closed symbols or 3000 PFU, open symbols). Naïve mice were challenged as a control (triangles). Symbols indicate the titers of lytic γHV68 virus detected in the lungs of individual mice at 3 and 6 dpi. The dotted line represents the limit of detection for the plaque assay used to measure viral titers. Chi-square analysis comparing numbers of mice with or without plaques detectable in lungs revealed significant differences (P < 0.05) only between the naïve mice challenged as a control and the other four groups.

It has been shown that mice latently infected with γHV68 are protected from a second, homologous viral challenge [22]. Therefore, as another way of assessing protective anti-viral immunity in aged γHV68-infected mice, we tested whether these mice could become re-infected with homologous virus. Young mice (3 months post infection) and aged mice (19 months post infection) were intranasally challenged with 400 or 3000 PFU γHV68, and lytic viral titers were monitored in the lung at 3 and 6 dpi (Figure 2). The data show high viral titers in young naïve mice at 3 and 6 dpi, as expected. However, only a low level of viral replication was detected at 3 dpi in the lungs of both young and aged latently infected mice and the level of viral replication did not vary with the dose of the challenge inoculum. By 6 dpi, virus was mostly cleared from the lungs of mice in both age groups. Similarly, there was no significant increase in viral titers in the spleens of either young or aged latently-infected mice after re-infection (data not shown). Together, the absence of viral recrudescence with increasing age and the comparable resistance of young and aged mice to re-infection with homologous virus suggest that there is no overall loss of immune control of γHV68 with age.

### Virus-specific CD8 T cell numbers are maintained and can mediate cytotoxicity in aged mice

**Figure 3:**
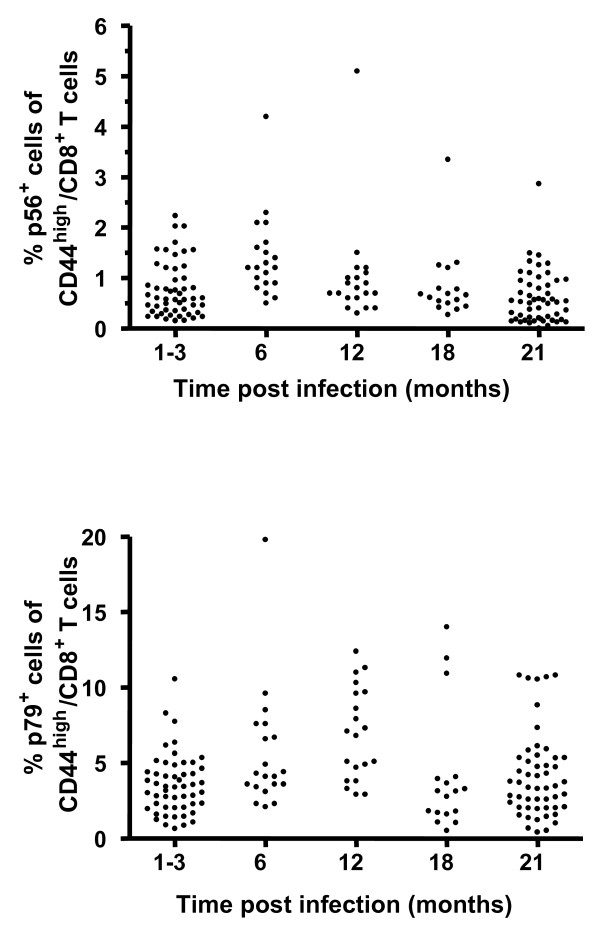
**Virus-specific CD8 T cell numbers are maintained with age**. Mice were bled at various times following γHV68 infection and virus specific CD8 T cells were detected using MHC class I tetramer staining and flow cytometry. Symbols represent the frequency of ORF6_487-495 _(p56, upper panel) and ORF61_524-531 _(p79, lower panel) specific cells among CD44^high ^CD8^+ ^T cells found in the peripheral blood of individual mice at the indicated times post infection.

**Figure 4:**
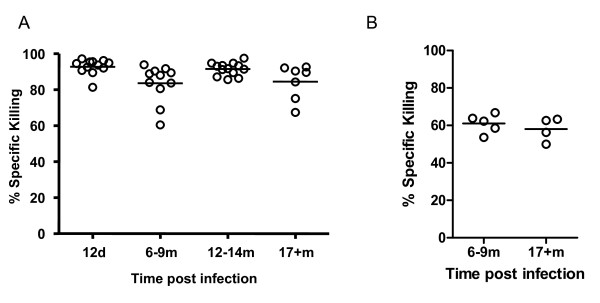
**Virus-specific CD8 T cells present in latently-infected mice retain CTL function with age**. Cytolytic activity of ORF61-specific CD8 T cells was measured at the indicated times post infection using a 16-17 h (Panel A) or a 4 h (Panel B) in vivo CTL assay as described in the *Materials and Methods*. Symbols represent specific killing calculated for individual mice. Bars indicate the medians calculated from data compiled from three independent experiments. Significance was assessed using the Mann-Whitney rank test (two-tailed, 95% confidence).

As it has been shown that both cellular and humoral immunity contribute to immune control of γHV68 [17,19,23-28], we sought to investigate the impacts of ageing on the functionality of each arm of the immune system during viral latency. In order to assess the impact of ageing on cellular immunity, we determined the numbers of γHV68-specific CD8 T cells present in latently-infected mice at different timepoints after infection using MHC class I tetramers specific for two well-characterized γHV68 epitopes p56 (ORF6_487-495_) and p79 (ORF61_524-531_)(Figure 3). The data show that p56- and p79-specific CD8 T cells are maintained in latently-infected mice over time, up to 21 months post infection. Although occasional individual mice were found to express higher than normal numbers of tetramer-positive cells as early as 6 months post infection, in general we did not observe an age-associated development of virus-specific T cell inflation as has been described for CMV infection [29-31], or virus-specific clonal expansions as we had previously observed in Sendai virus-infected mice [32]. We also assessed the cytolytic activity of the ORF61-specific CD8 T cells present in latently-infected mice at various timepoints following infection using an *in vivo *CTL assay. As shown in Figure 4, the cytolytic activity against ORF61-pulsed targets measured in individual mice remained stable for up to two years after infection. In addition, in agreement with a previous report [33], the numbers and CTL function of γHV68-specific CD8 T cells specific for ORF-6-pulsed targets were also maintained with age (data not shown).

### Virus-specific antibody levels are maintained, but neutralizing activity declines

**Figure 5:**
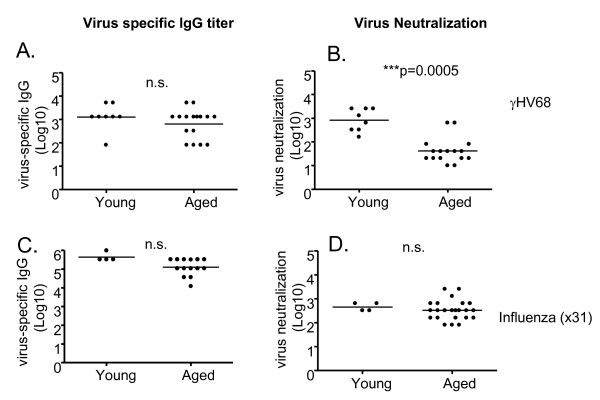
**Serum titers of γHV68-specific antibody are maintained in latently infected mice with age, but serum neutralization activity wanes**. The titers of virus-specific IgG were measured in the serum of latently-infected young (1-3 months p.i.) and aged (18-22 months p.i.) by ELISA (Panel A). Neutralization activity (measured in the same sera samples as A) were determined using an in vitro neutralization assay (Panel B). As a control, antibody titers (C) and neutralization activity (D) were measured in serum taken from mice (young and aged, as above) previously infected with influenza virus ×31. Symbols represent serum antibody titers and neutralization activities measured for individual mice. Bars indicate the medians calculated from the data shown. Significance was assessed using the Mann-Whitney rank test (two-tailed, 95% confidence). Ns; not significant.

**Figure 6:**
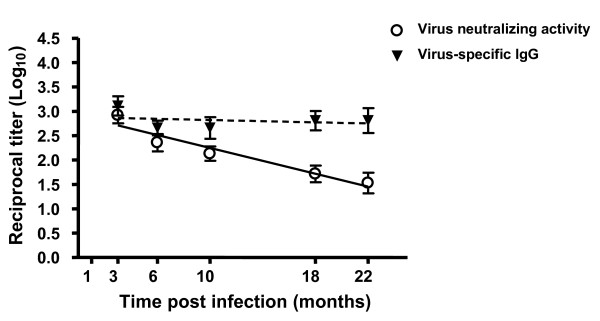
**Neutralizing titers of γHV68 decline progressively with time after infection**. Serum titers of γHV68-specific IgG (closed triangles) and neutralizing antibody (open circles) were measured in individual mice at the indicated months post infection. Symbols represent the mean reciprocal titers (log_10_), ± standard deviation, calculated at each timepoint (≥ 8 mice analyzed per timepoint). A linear regression was performed analyzing time versus reciprocal titer for virus-specific IgG or neutralizing antibody. The slope for virus specific IgG did not differ significantly from zero (p = 0.6780), however the slope for virus neutralizing antibody did (p = 0.0085).

**Figure 7:**
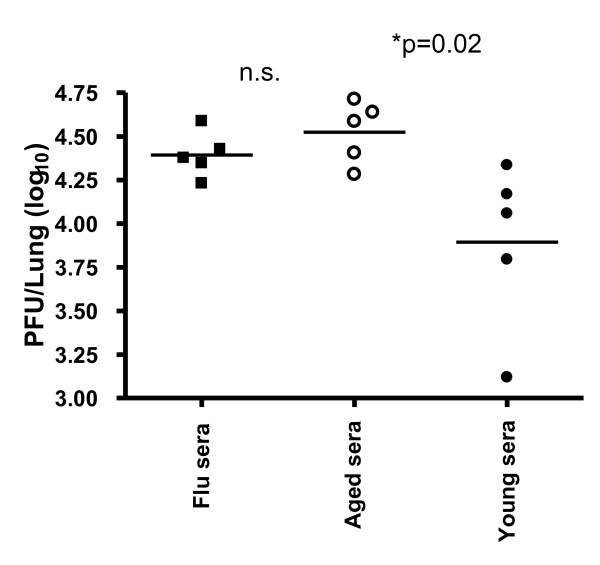
**Sera from aged mice is less protective in vivo against acute γHV68 infection**. One hundred microliters of sera from young (3 months p. i.) or aged (22 months p. i.) γHV68-infected animals was injected intravenously into naïve C57BL/6 mice one day prior to intranasal γHV68 infection (400 PFU). Control mice received convalescent sera from influenza virus-infected mice. Symbols represent lung viral titers measured in individual mice at 5 days post infection using a standard plaque assay. Bars indicate the medians calculated from the data shown. Significance was assessed using the Mann-Whitney rank test (two-tailed, 95% confidence). Ns; not significant

We next determined the impact of ageing on humoral immunity. It has previously been shown that following intranasal infection with γHV68, virus-specific IgM antibodies peak on day 10 and subsequently subside, and class-switched antibody levels increase over the first 3 weeks of infection and are then maintained at constant levels for at least 90 days [34,35]. As shown in Figure 5A, we found that the serum titers of virus-specific IgG were similar between recent (1-3 months p.i.) and long-term (18-22 months p.i.) latently infected mice. However, serum neutralization titers were found to be greatly reduced in the majority of mice 18-22 months postinfection (Figure 5B). The reduction in neutralizing activity was an unexpected result, as humoral immunity has been shown to be remarkably long-lived for other viruses, including small pox and influenza virus [36-38]. This loss of neutralizing activity with age was specific for γHV68 infection, as serum antibody titers and neutralizing activity were maintained long-term in influenza virus-infected mice (1-3 and 18-22 m.p.i; Figure 5C, D). Interestingly, the loss of neutralizing activity was not restricted to aged mice, rather the decline was gradual over the course of γHV68 infection (Figure 6). Importantly, the decline in neutralizing titer had functional implications, as passively-transferred sera from aged γHV68-infected mice was less efficient in protecting naïve mice from de novo γHV68 challenge than sera transferred from young γHV68-infected mice (Figure 7). Taken together, whereas we found no defect in T cell immunity or loss of overall immune control of latency, there was a clear age-associated change in humoral immunity over the course of latent γHV68 infection.

### Aged mice can control de novo infection

**Figure 8:**
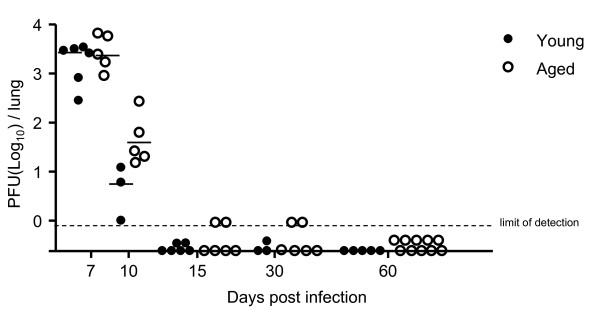
**Aged mice clear lytic virus from their lungs following de novo γHV68 infection with only slightly delayed kinetics**. The titers of lytic virus in the lungs of individual young (3 months old, closed symbols) and aged (19-24 months old, open symbols) C57BL/6 mice at the indicated days following de novo intranasal γHV68 infection (400 PFU) were measured using a standard plaque assay. The dotted line indicates the plaque assay's limit of detection.

**Figure 9:**
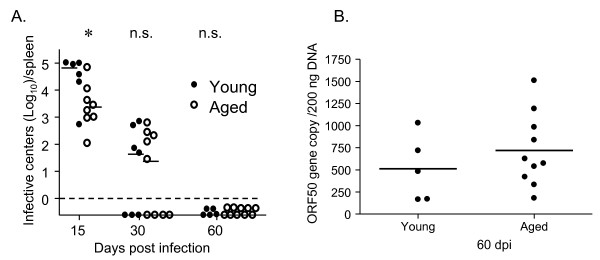
**Aged mice are capable of maintaining control over latent virus following de novo γHV68 infection**. The levels of latent virus present in the spleens of γHV68-infected young (3 months old, closed symbols) and aged (19-24 months old, open symbols) mice at the indicated days post infection were measured using an infective center assay (Panel A) and a genome copy assay (Panel B), as described in the *Material and Methods*. The dotted line indicates the limit of detection of the infective center assay. Each symbol represents data obtained from an individual mouse and bars indicate the means calculated from the data shown.*; P ≤ 0.05 as determined using the Student's t-test. n.s.; not statistically significant.

**Figure 10:**
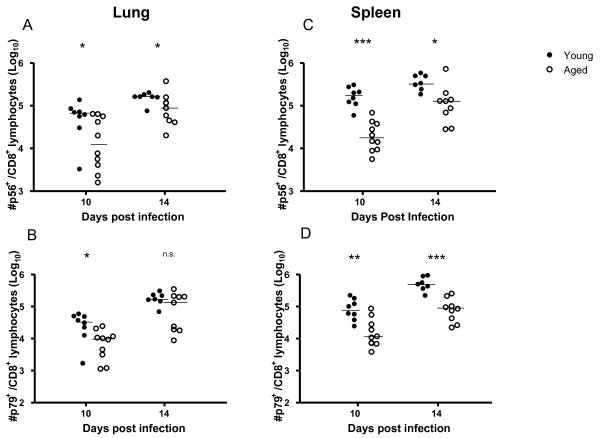
**Virus-specific CD8 T cells are generated in aged mice following de novo γHV68 infection**. MHC class I tetramer staining and flow cytometric analyses were performed to determine the absolute numbers of ORF6/p56- and ORF61/p79-specific CD8 T cells present in the lungs (A, B) and spleens (C, D) of C57BL/6 mice infected when young (3 months old, closed symbols) or aged (20 months old, open symbols) at the indicated days following de novo γHV68 infection. Symbols represent data obtained from individual mice and bars indicate the medians. Significance was assessed using the Mann-Whitney (two-tailed, 95% confidence). Ns = not significant, * = P ≤ 0.05, ** = P ≤ 0.01, *** = P ≤ 0.001.

Despite the decline in neutralizing antibody titers, our data indicate that T cell function is intact and immune control over γHV68 latency is maintained with ageing. This observation is consistent both with our previous finding that T cells can control reactivation in the absence of neutralizing antibodies [19] and the notion that T cell memory generated when young retains function into age [39,40]). However, de novo infection in the absence of CD4 help has been shown to generate defective CD8 T cell memory [41-43], and the degree of CD4 dependence may vary with the pathogen. Specifically for γHV68, it has been shown that CD4-deficient mice are able to clear lytic virus and viral latency is established normally, however these mice progressively lose immune control over viral latency resulting in recrudescence of lytic virus around 42 dpi [17]. Because there is a well-established age-associated decline in CD4 T cell function [44-49], we examined the ability of aged naïve mice to control de novo infection as another measure of the impact of ageing on functional γHV68-specific immunity. The data show that clearance of lytic virus from the lungs was only slightly delayed (not statistically significant) in aged mice compared to young mice following the initial infection (Figure 8) and that comparable, stable levels of latent virus were detected in the spleens of both groups of mice, as assessed by both infective center and genome copy assays (Figure 9). However, analysis of γHV68-specific CD8 T cell responses elicited in infected mice using MHC class I tetramers revealed generally reduced numbers of virus-specific p56 and p79 CD8 T cells in aged compared with young mice after de novo infection (Figure 10).

**Figure 11:**
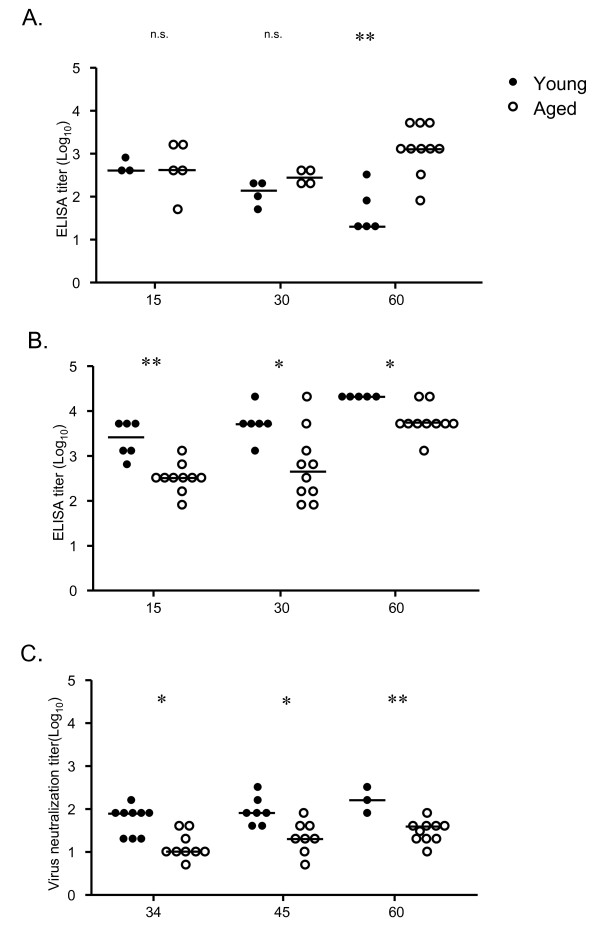
**Aged mice produce reduced serum titers of class switched antibody and reduced neutralization titers following de novo γHV68 infection**. Serum titers of γHV68-specific IgM (A) and IgG (B) were measured by ELISA in young (3 months old, closed symbols) and aged (19-24 months old, open symbols) C57BL/6 mice at 15, 30, and 60 days post infection. (C) The virus neutralization activity of sera at 34, 45, and 60 days post infection was determined as described in the *Materials and Methods*. Each symbol represents data obtained from an individual mouse and bars indicate the medians calculated from the data shown. Statistical significance was determined using the Mann-Whitney test.**; P ≤ 0.01. *; P ≤ 0.05.

Analysis of virus-specific serum antibody titers at 15, 30 and 60 dpi showed that although titers of virus-specific IgM were comparable in young and aged mice early after infection (days 15 and 30), virus-specific IgM titers were increased in aged mice at 60 dpi (Figure 11A), consistent with an age-associated deficiency in the generation of class-switched antibodies at all timepoints examined (Figure 11B). In addition, serum neutralization titers were significantly compromised in aged mice at all timepoints examined (Figure 11C). Taken together, despite statistical differences in cellular and humoral immunity after infection of aged compared with young mice, the aged mice were able to control infection comparably to young mice (Figures 8 and 9).

## Discussion

The impaired ability of the elderly to control acute primary virus infections is well-characterized, whereas less is known about the impact of age-associated decline in immunity in control of latent, persistent and/or chronic viruses. Here we have exploited a robust and well-characterized mouse γ-herpesvirus model to show that there is no overt loss of immune control of latent virus with ageing. The experimental mouse model has allowed us to separately dissect the impact of ageing on cellular and humoral mechanisms of immune control. Interestingly, the data show differential impacts of ageing on cellular and humoral immunity- numbers and function of virus-specific CD8 T cells were maintained, whereas there was a progressive decline in viral neutralizing activity, despite maintenance of virus-specific antibody titers.

The impact of ageing on the immune control of the human γ-herpesviruses has not been well-studied. One report showed increased numbers, but decreased function, of CD8 T cells specific for an immunodominant EBV epitope [50], and another report showed higher levels of reactivation with ageing [51]. A third report showed increased anti-viral antibodies in the elderly, consistent with enhanced reactivation [52]. Initially, these data appear to be in contrast to our results using the γHV68 mouse model, in which we showed no decline in cellular immune control of γHV68 and no reactivation or increased latent load with ageing. However, despite anecdotal reports of declining immune control of EBV with ageing cited above, a strong correlation between ageing and human γ-herpesvirus-associated malignancies has not been demonstrated. There are a few notable exceptions- the age-associated development of non-HIV-associated Kaposi's sarcoma [9], the bimodal age distribution of EBV-associated Hodgkin's Disease with an increased frequency among the elderly [53], and a recently-identified, age-related EBV-associated B cell lymphoproliferative disorder [54-57]. However, as EBV infects > 90% of the human population, one would expect a more dramatic increase in the incidence of γ-herpesvirus-associated malignancies in the elderly if ageing resulted in failure to control viral latency. Thus, our data in the mouse model showing no striking loss of immune control of γHV68 with ageing are consistent with the absence of a strong increase in γ-herpesvirus associated malignancies in man.

However, an important caveat to our studies is that the experimental mice were maintained under specific pathogen free conditions during aging. This, of course, does not reflect the human situation, as humans are exposed to multiple acute and chronic infections as they age. It is possible that intermittent stimulation elicited by heterologous infections would disrupt quiescent latency and reveal differences in the ability of young and aged individuals to control the latent infection. Despite this experimental caveat, our studies in mice taken together with the observations in humans suggest that ageing has little effect on the immune control of γ-herpesvirus latency. These data are also consistent with the idea that T cell memory generated when young is well maintained [39,40], and with the data reported here and elsewhere [33] showing that there is no decline in function of γHV68 CD8 T cells with ageing.

Our conclusion is further supported by the findings that γ-herpesvirus-associated malignancies increase dramatically in instances where immune function is severely compromised. Cellular immunosuppression is a frequent therapy following transplantation or for autoimmune disease. It is also the consequence of some secondary viral infections including HIV. Importantly, during cellular immunosuppression, antibody titers are not immediately affected and humoral immunity serves as an essential back-up mechanism. There are several examples of γ-herpesvirus-associated pathology during immunosuppression. First, decline in T cell function as a consequence of HIV infection is attributed to the development of Kaposi's sarcoma in KSHV-infected individuals [9]. Second, EBV-associated lymphoproliferative disease is a well-known complication of immunosuppressive therapy after stem cell or solid organ transplantation [58], which is becoming increasingly frequent in elderly populations. Susceptibility to develop lymphoproliferative disease following transplant immunosuppression has been correlated with EBV latent load or EBV copy number in the latently-infected cell [59-61]. A specific link between defects in humoral immunity under conditions of impaired cellular immunity is suggested by the correlation between the development of Kaposi's sarcoma in KSHV-infected individuals and reduced levels of neutralizing antibody [62]. Third, immunosuppressive therapy for autoimmune diseases such as rheumatoid arthritis and psoriasis, which are prevalent in the elderly, is frequently complicated by EBV-associated lymphoproliferative disease and lymphoid neoplasms [63-65]. Thus, with loss of T cell immunity due to disease or therapeutic intervention, an impact of ageing on humoral immunity may have consequences for control of γ-herpesvirus latency.

Evidence for an age-associated decline in one arm of immunity is difficult to detect in the intact host. For example, in the face of functional cellular immunity, consequences of a decline in humoral immunity would not be readily observed. The experimental mouse model has made it possible to assess the function of the two arms of adaptive immunity separately. Although CD8 T cells play a central role in maintaining control of latency, CD4 T cells and antibody also make important contributions [11,12]. It is clear that γ-herpesvirus latency is controlled at multiple levels and that the availability of a mouse model for analysis of viral control and anti-viral immunity in ageing mice provides a powerful experimental approach.

The declining neutralizing activity of γHV68-specific antibodies with time after infection was an unexpected result. Despite the short half life (< 3 weeks) of antibodies [66-68], humoral immunity to viruses is usually long-lived [69,70]. For example, vaccinia-specific antibody responses can be identified as long as 75 years after a single vaccination [71]. The longevity of the antibody response is due to the development of long-lived antibody secreting plasma cells that reside in the bone marrow [72,73]. These cells have a half-life of ~140 days, and are replenished at a low rate by differentiation of memory B cells [72]. Recently, it was determined that survivors of the 1918 influenza virus pandemic had circulating B cells that secreted neutralizing antibodies to the virus [38]. Our data show a striking decline in the neutralization activity of γHV68-specific antibodies with time after infection, despite sustained titers. This is a novel and unexpected observation, which has significant implications for ageing immunology research and vaccination strategies, and merits further study. Changes in EBV-specific antibodies throughout latency have been previously reported. Specifically, there are age-associated changes in patterns of specificity of anti-EBV antibodies [74], that have been shown to correlate with changes in the latent state of the virus [75]. Titers of EBV antibodies to some epitopes are maintained or increase with age, but the antibodies are of low avidity and function poorly [52]. It is possible that changes in the expression of γ-herpesvirus epitopes over time, perhaps due to changes in immune control of latency, would be manifest in changes in antibody specificities. Concerning γHV68, we hypothesize that the gradual decline in neutralizing activity is either a consequence of changes in viral gene expression during long-term latency or reflective of an increased propensity toward viral reactivation over time, both of which would result in the presentation of new epitopes to drive de novo antibody responses. Also, because the progressive decline in neutralizing activity was observed in γHV68-infected mice and not influenza-infected mice, this phenomenon is likely associated with the nature of the infection rather than the biology of ageing.

A defect in the ability of aged individuals to mount an effective de novo antibody response is well-described [76-80]. Underlying B cell defects associated with ageing include decreased germinal center formation, decreased levels of somatic mutation and the production of poorly protective antibodies [81-85]. The age-related defects are dependent largely on defective CD4 T cells rather than inherent age-associated defects in B cells. For example, the generation of germinal centers is dependent on CD4 T cell cognate helper function, which is reduced with age [86]. Our data describing the development of sub-optimal humoral immunity following de novo γHV68 infection of aged mice is consistent with an age-associated CD4 T cell defect. Interestingly, however, the CD8 T cell effectors appeared to be fully functional and did not permit recrudescence of lytic virus as long as 60 days after infection, in contrast to the results previously shown for CD4-deficient mice, which cannot maintain control of the virus and undergo viral recrudescence as early as 42 days post infection.

## Conclusion

We have found that immune control of a latent mouse γ-herpesvirus is not severely impaired with age. In addition, aged mice can control a de novo γHV68 infection with only slightly delayed viral clearance, and maintain control of latency. These data are consistent with the lack of an increased incidence of γ-herpesvirus-associated malignancies in the elderly. However, immunosuppression of the elderly is clearly correlated with increased onset of malignancies and may reflect consequences associated with age-related changes in humoral immunity.

## Methods

### Mice

Young (6-8 weeks) and aged (≥ 18 months) C57BL/6 mice and B6.SJL-Ptprc^a^Pepc^b^/BoyJ (B6.SJL, CD45.1) congenic mice were obtained from the Trudeau Institute animal breeding facility. All mice were housed under specific pathogen free conditions before and after infection. All animal procedures were approved by the Institutional Animal Care and Use Committee at the Trudeau Institute.

### Virus Stocks, Infections and Vaccination

Clone WUMS of γHV68 [14] was propagated in NIH-3T3 cells. Virus titers were determined by plaque assay on NIH-3T3 cells. Influenza virus A/HK-x31 (×31, H3N2) was grown, stored, and titrated as described previously [87]. Female mice were anesthetized with 2,2,2-tribromoethanol (200 mg/kg) prior to intranasal (i.n.) infection with 400 PFU γHV68 or 300 EID_50 _of A/HK-x31 influenza virus.

### MHC Class I Tetramer Staining

Peripheral blood (100-200 μl), obtained by nicking the tail, was collected in PBS containing 10 U/ml heparin (Sigma-Aldrich, St. Louis, MO). Single cell suspensions from the spleens of individual mice were prepared by mechanical disruption and straining through nylon mesh, and lung cells from individual mice were processed with collagenase D. Samples were depleted of erythrocytes by treatment with buffered ammonium chloride solution. Lymphocytes were enriched from lung cell suspensions by Percoll gradient centrifugation (Hogan, 2002) and from spleen cell suspensions by panning against goat anti-mouse IgG (Jackson ImmunoResearch Laboratories, West Grove, PA). Isolated cells were incubated with Fc block (BD Biosciences, San Jose, CA) for 15 minutes on ice, and then stained with PE- or APC-labeled MHC class I tetramers specific for γHV68 ORF6_487-495_/D^b ^or ORF61_524-531_/K^b ^for one hour at room temperature. All tetramers were generated by the Molecular Biology Core Facility at the Trudeau Institute, as previously described (Altman, 1996). Tetramer-labeled cells were then stained with fluorescently-conjugated antibodies specific for mouse CD8α and mouse CD44 (BD Biosciences) for 30 minutes on ice, washed, and then fixed in 1% paraformaldehyde. Data were collected on a Becton Dickinson FACSCalibur flow cytometer (BD Biosciences) and then analyzed using FlowJo software (Tree Star, Inc., Ashland, OR).

### Plaque Assay

The concentration of lytic virus in lung tissue was determined using a standard plaque assay on NIH-3T3 mouse fibroblasts [17]. Lung tissue obtained at various times post infection was mechanically homogenized and serial-dilutions of the homogenates were prepared. Diluted homogenates were incubated on monolayers of 3T3 cells for one hour at 37°C/10% CO_2_, after which the monolayers were overlaid with carboxymethyl cellulose (Sigma-Aldrich) and incubated again at 37°C/10% CO_2_. Six days later, monolayers were fixed with methanol, stained with 8% Giemsa stain, and plaques counted.

### Infective Center Assay

The frequency of latently infected cells capable of spontaneous in vitro reactivation was assessed using an infective center assay, as previously described [88]. 10-fold serial dilutions (in triplicate) of splenocytes starting at 10^6 ^cells/well were plated onto monolayers of NIH-3T3 mouse fibroblast cells. Monolayers were incubated overnight at 37°C and then overlaid with carboxymethyl cellulose. Plaques were quantitated 6 days later after methanol fixation and Giemsa staining. Samples were also assayed following one cycle of freeze/thaw to determine the contribution of lytic virus to the overall viral titers. The number of latently infected cells was then calculated as the difference between the total number of infected cells and the number of lytically infected cells.

### Limiting-dilution PCR Analysis

The frequency of cells carrying viral genome was determined using a limiting dilution nested PCR assay (LDA/PCR) for the γHV68 open reading frame 50 (ORF50) gene as described [19,89]. Briefly, CD19^+ ^B cells were purified from the spleens of infected mice as previously described [90]. B cells were resuspended in isotonic buffer and starting at 10^4 ^or 10^5 ^cells/well, were diluted with uninfected NIH-3T3 cells, and then transferred into 96 well plates. Twelve replicate reactions were performed for each cell dilution per experiment. Subsequently, the ORF50 gene was amplified from cell lysates with two rounds of nested PCR. The final PCR product was electrophoresed on a 3% agarose gel and stained with ethidium bromide. The reciprocal frequency of cells carrying viral genome was determined using linear regression with a 95% degree of confidence.

### Determination of genome copy

Genome copy was estimated as previously described [21], with the following changes. DNA was extracted from 5 × 10^6 ^B cells using a DNeasy kit (Qiagen), according to manufacturer's protocol. ORF50 gene copy number was determined for 200 ng of DNA per sample, using a standard curve quantitation method on an Applied Biosystems 7500 Real-Time PCR System (Applied Biosystems, Foster City, CA). Reactions were run in duplicate, and naïve C57BL/6 splenocyte DNA was used as a negative control. A low ROX AB Taqman Gene Expression master mix (Applied Biosystems, Foster City, CA) was used, providing repeatable detection of copy numbers as low as 3 per 200 ng of DNA. Primers, probes, and reaction cycles used are described [21].

### Enzyme-Linked Immunosorbent Assay (ELISA)

Virus specific IgG titers in sera were determined by ELISA [19]. Nunc ImmunoMaxisorp plates were coated with purified virions at a concentration of 0.5 μg/well. Following an overnight incubation at 4°C, plates were washed with PBS-Tween (0.05%), and subsequently blocked with PBS/BSA (3%) overnight at 4°C. Dilutions of sera, starting at 1/20, were prepared in PBS/0.05%Tween/0.5%BSA and added to the antigen coated plates. After an overnight incubation at 4°C, virus-specific IgG was detected using alkaline phosphatase-conjugated goat anti-mouse IgG antibody (Sigma-Aldrich) and p-nitrophenyl phosphate (Sigma-Aldrich). Optical densities were read at 405 nm using a VMax^® ^microplate reader (Molecular Devices, Sunnyvale, CA). Serum antibody titers are expressed as the highest reciprocal dilution of serum giving an OD_405 _reading at least two times greater than the normal mouse serum control.

### Virus Neutralization Assays

For the γHV68 neutralization assay, virus (10^2 ^PFU) was incubated with serial dilutions of heat-inactivated serum samples in 96-well plates for 1 h at 37°C. Following the incubation, virus/sera mixtures were transferred to 96 well flat-bottom plates containing monolayers of 3T3 cells. After 7 days of incubation at 37°C, 10% CO_2_, the monolayers were fixed and plaque formation revealed by staining with 5% Giemsa stain. Neutralization titers are expressed as the highest reciprocal dilution of sera required to cause a 50% reduction in CPE (cytopathic effect).

For the influenza virus neutralization assay, ×31 virus (100 EID_50_/well) was incubated with serial dilutions of heat-inactivated sera, prepared in Zero-Serum media (Diagnostic Hybrids, Athens, OH) containing 4 μg/ml trypsin, in 96-well round-bottom plates for 1 h at 37°C. Following the incubation, virus/sera mixtures were transferred to 96 well flat-bottom plates containing monolayers of MDCK cells. Plates were then centrifuged at 800 × *g *for 1.5 hrs. Following centrifugation, the virus/sera mixtures were removed, fresh media added to each well, and the plates incubated at 33°C, 10% C0_2 _for 14-16 hours. Monolayers were fixed in 80% acetone and infected cell foci were revealed by incubating with a combination of biotinylated mouse anti-influenza A monoclonal antibodies (Millipore, Billerica, MA), alkaline-phosphatase conjugated goat anti-mouse IgG (Sigma-Aldrich), and Sigma-Fast BCIP/NBT substrate (Sigma-Aldrich). Neutralization titers are expressed as the highest dilution of sera prior to which the number of foci observed is equivalent to the number of foci counted in normal mouse sera/virus-only control wells.

### Serum transfer

Serum obtained from latent γHV68-infected young (2-4 months post infection) and aged (18-24 months post infection) C57BL/6 mice was administered intravenously to young, naive C57BL/6 mice one day prior to intranasal γHV68 infection (400 PFU). As a control, mice were given normal mouse serum or convalescent serum obtained from C57BL/6 mice previously infected with influenza A/PR8/34 virus (300 EID_50_). Lungs were harvested from recipient mice on day 5 post infection and viral loads were measured using the standard γHV68 plaque assay.

### In vivo CTL assay

Target splenocytes harvested from B6.SJL (CD45.1^+^) congenic donor mice were incubated with peptides (10 μg/ml) at 37°C in 10% CO_2 _for 5 h with occasional mixing. The peptides included γHV68 ORF61_524-531 _and an irrelevant peptide (either influenza NP_366-374 _or Sendai NP_324-332_). Cells were washed and labeled with 2.5 μM or ≤ 0.5 μM of 5,6-carboxyfluorescein diacetate succinimidyl ester (CFSE; Molecular Probes, Eugene, OR) to obtain CFSE^hi ^(ORF61_524-553_) and CFSE^low ^(negative control peptide) groups. Following CFSE labeling, cells were combined at equal ratios, washed three times in PBS, and resuspended at a final concentration of 2 × 10^8 ^cells/ml. Twenty million cells (100 μl) were injected intravenously into C57BL/6 mice (CD45.2^+^) previously infected with γHV68, or naïve mice as a negative control. At 4 h or 16-17 h post transfer, spleens were harvested from recipient mice and single cell suspensions were prepared for flow cytometric analyses as described above. The individual populations of peptide-pulsed donor CD45.1^+ ^cells present in the spleens of recipient mice were identified and enumerated using a combination of APC-conjugated antibodies specific for mouse CD45.1 (eBioscience, San Diego, CA) and CFSE staining intensity. Percent specific lysis was calculated using the following formula: [1-(ratio uninfected/ratio infected)] × 100, where "ratio" refers to the percentage of irrelevant peptide-pulsed cells divided by the percentage of relevant peptide-pulsed cells.

### Statistical Analyses

Where indicated, groups were compared statistically using the Student's t-test, the nonparametric Mann-Whitney rank test, or linear regression, or the Chi-square test. P values ≤ 0.05 were considered significant. All statistical analyses were performed using Prism software (GraphPad, La Jolla, CA).

## Competing interests

The authors declare that they have no competing interests.

## Authors' contributions

EJY, I-JK, MLF, KGL and CEB participated in the design of the study and in performing the experiments. TC contributed to the in vivo CTL analysis. DLW contributed to the discussion of the results and the writing of the manuscript. MAB conceived the study, directed the experimentation and drafted the manuscript. All authors read and approved the final manuscript.
